# Three-dimensional cage-like microscaffolds for cell invasion studies

**DOI:** 10.1038/srep10531

**Published:** 2015-05-27

**Authors:** Barbara Spagnolo, Virgilio Brunetti, Godefroy Leménager, Elisa De Luca, Leonardo Sileo, Teresa Pellegrino, Pier Paolo Pompa, Massimo De Vittorio, Ferruccio Pisanello

**Affiliations:** 1 Center for Biomolecular Nanotechnologies, Istituto Italiano di Tecnologia, Via Barsanti, 73010 Arnesano (Lecce), Italy; 2 Dipartimento di Ingegneria dell’Innovazione, Università del Salento, via per Monteroni, 73100 Lecce, Italy; 3 Nanochemistry Department, Istituto Italiano di Tecnologia, via Morego 30, 16163 Genova, Italy

## Abstract

Cancer cell motility is one of the major events involved in metastatic process. Tumor cells that disseminate from a primary tumor can migrate into the vascular system and, being carried by the bloodstream, transmigrate across the endothelium, giving rise to a new tumor site. However, during the invasive process, tumor cells must pass through the extracellular matrix, whose structural and mechanical properties define the parameters of the migration process. Here, we propose 3D-complex cage-like microstructures, realized by two-photon (TP) direct laser writing (DLW), to analyze cell migration through pores significantly smaller than the cell nucleus. We found that the ability to traverse differently sized pores depends on the metastatic potential and on the invasiveness of the cell lines, allowing to establish a pore-area threshold value able to discriminate between non-tumorigenic and tumorigenic human breast cells.

The mechanisms underlying cell migration on two-dimensional surfaces have been well characterized in the past years. By virtue of polarized intracellular signaling, cells extend actin-rich protrusions, generate new focal adhesion points, retract their trailing edge and displace the cell body^1^, giving rise to the so-called motility cycle^2^. *In vivo*, however, cells are exposed to a complex three-dimensional (3D) multifunctional environment, whose chemical and mechanical properties influence (and are influenced by) cell motility^3^,^4^,^5^. Although there is clear evidence that 3D environments better simulate cell physiological conditions, it is also expected that extracellular matrix texture organization can promote or prevent migration^6^,^7^,^8^. The main advantage of DLW-fabricated structures compared to fibrous assemblies commonly used to simulate *in vivo* extracellular environments^9^ (e.g. collagen, Matrigel and fibrinogen) is the possibility to control matrix architecture as well as pore and mesh size with a resolution of tens of nanometers. In 2008 P. Tayalia *et al.*^10^ have shown that TP-engineered woodpile structures can be used to induce and track migration of fibrosarcoma cells within a well-controlled 3D environment, resulting in increased cell speed if compared to 2D substrates. These findings gave impetus to a series of *in vitro* studies, resulting in new insights in cell mechanics^11^,^12^ and in the influence of extracellular environment on cell differentiation^13^. Very recently, Greiner *et al.*^14^ have used a highly resolved cell-sieve to study the influence of nuclear stiffness on the motility of epithelial cells and mouse embryonic fibroblasts, showing the potential of this technology when coupled to controlled chemoattractive signals. The interaction between DLW-fabricated scaffolds and a wide variety of cell lines, including neuron-like cells, has been investigated so far^6^,^10^,^11^,^14^,^15^, but none of these structures has been shown to be able to distinguish between different cell populations based on their invasive potential. Moreover, in tumor cell migration studies, one of the main challenges is to have analytical tools that allow correlating the *in vitro* tumor cell migration with their *in vivo* invasiveness and metastatic ability. Boyden chambers^16^,^17^,^18^ and similar commercial kits, commonly used as *in vitro* invasion assays, evaluate the ability of cells to migrate through a porous film (~8 μm in diameter) coated by a matrix of basement membrane compounds^19^,^20^. Findings obtained with these systems are in agreement with recent observations on *in vitro* models for tumor cell extravasation, where tumorigenic cell lines were observed emitting protrusions across the endothelium and creating openings as small as ~8 μm to allow for nuclear transmigration^13^,^21^,^22^. Unfortunately, tumor cell lines that do not migrate in the Boyden chamber assay are instead metastatic *in vivo*, thus providing false negative results^21^,^23^,^24^,^25^,^26^,^27^.

Here we propose 3D cage-like microstructures allowing cell invasion only by cellular deformation and translocation through a porous mesh monolayer. Non-malignant and malignant cell lines derived from the human breast were used to statistically evaluate the percentage of invaded structures as a function of pore area: the obtained data suggest the existence of a pore-size threshold value below which only tumor cells are able to pass. This is confirmed also by co-culture experiments, in which tumor cells were still found to be able to traverse small pored meshes. Moreover, since it is well known that the acto-myosin cytoskeleton plays a crucial role in cell migration and invasion processes^28^,^29^,^30^,^31^, we analyzed the myosin distribution in cells contacting and/or invading the structures.

## Results

### Cage-like microscaffolds

**Figure 1:**
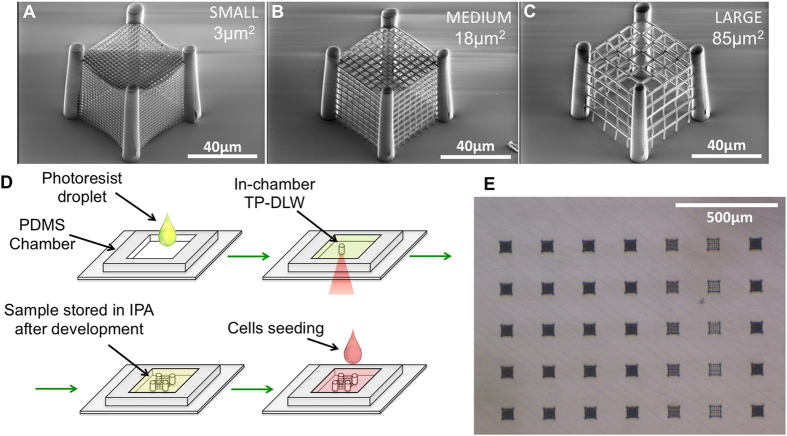
Cyanamide biosynthesis from L-canavanine. ** Panels (A–C)** SEM bird eye views of cage-like structures having ~3, 18, and 85 μm^2^ pore-area. **Panel (D)** Steps of the fabrication process. From left to right and from top to bottom: (i) a drop of IP-L photoresist is dropcasted into a PDMS chamber previously glued on a glass microscope coverslip, (ii) the resist is exposed by means of TP-DLW, (iii) sample is developed and stored in IPA and (iv) cells are seeded within the chamber. All realized cages were kept in liquid ambient to avoid any deformation induced by solvent evaporation, apart for the samples used for SEM inspection. **(Panel E)** Representative optical microscope image of a sample after development.

Four 60 μm-high pillars with 10 μm diameter were realized to support five mesh monolayers, describing a total cage volume of about 50 μm × 50 μm × 50 μm. As displayed in the representative SEM images reported in Fig. 1A–C, meshes with three different pore sizes were investigated, namely ~3, 18, and 85 μm^2^, hereafter referred to as *small* (S), *medium* (M) and *large* (L) pores, respectively.

To fabricate these cage-like structures, a droplet of commercially available and biocompatible negative tone photoresist (2-(Hydroxymethyl)-2-[[(1-oxoallyl)oxy]methyl]-1,3-propanediyldiacrylate, known as IP-L from Nanoscribe GmbH, see viability test in Figure S1) was cast into a polydimethylsiloxane (PDMS) chamber previously glued on a 170 μm-thick microscope coverslip (the fabrication process flow is schematized in Fig. 1D). IP-L was exposed by a Nanoscribe two-photon lithography system equipped with a femtosecond-pulsed laser at 780nm and a high numerical-aperture oil-immersion objective (N.A. = 1.4, see experimental section for more details). Writing time for each structure was optimized to ~30 min, and after development the samples were stored in liquid (isopropyl alcohol) in order to avoid structural modifications induced by surface tensions caused by solvent evaporation. To show the ability of this design in estimating cell invasive potential, three different human cell lines derived from breast epithelium were chosen as representative specimens because of their different invasive properties: (i) MCF10A, non-tumorigenic, derived from mammary gland; (ii) MCF7, derived from primary breast ductal carcinoma and (iii) MDA-MB-231, derived from adenocarcinoma metastatic site. Each cell line was seeded into a PDMS chamber in which several cages with S, M and L pores were previously realized (see Fig. 1E and Figure S2). Samples were fixed when the confluence point was reached (culturing time varied from 48 to 96h depending on the cell line) and DAPI-based nuclei staining was performed (see Figure S3 and Figure S4 for bright field images of representative samples just after seeding and at confluency). Figure S5 highlights how all three cell lines approach the cages, often emitting very long protrusions already after 24 h from seeding, letting us suggest that the early stages of cage approaching are mostly the same for MCF10A, MCF7 and MDA-MB-231. However, because of the different doubling times of the analyzed cell lines, and given that even after 48 h from cell seeding the cell distribution is not homogeneous, we have chosen to stop the experiment when cells reached confluency. High-resolution 3D-confocal microscope analysis was then carried out in liquid environment by detecting luminescence from the bottom of the structures (see experimental section for details).

### 3D confocal analysis

**Figure 2:**
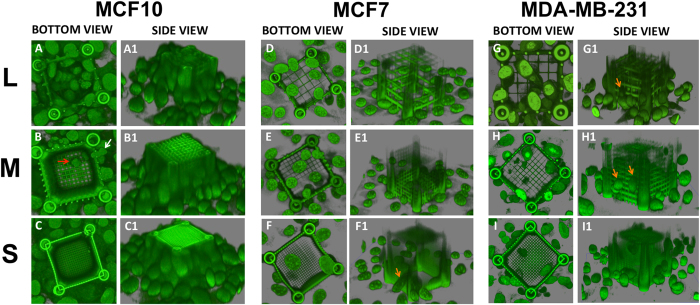
3D Confocal reconstructions of representative cage-cell interactions for each pore size. ** Panels (A–C)** were obtained for MCF10A cell line, **Panels (D–F)** for MCF7 and **Panels (G–I)** for MDA-MB-231. All images were obtained after DAPI staining on fixed culture in PBS. All images are in false colour and microsctructures have a broad emission spectrum. Their luminescence can be collected together with the one of DAPI stained cell nuclei, in the range [420 nm, 500 nm].

As shown by the bottom and side views of the 3D confocal reconstructions (Fig. 2A,B), MCF10A cells were able to cross both L and M pores. The invasion took place along all the spatial directions: cells can deform their nuclei passing through the side meshes or going down from the top (see cells indicated by white and red arrows in Fig. 2B, respectively). Although also S-sized cages were approached from all directions by MCF10A, they remained empty (Fig. 2C). In the latter case, MCF10A cells tend to accumulate all around the cage by virtue of their spontaneous ability to form acini-like structures in confluent cultures mimicking their *in vivo* architecture^32^ and keeping their nuclei outside the S-cages (Fig. 2C).

Remarkably, both investigated tumor cell lines were able to pass through all pore sizes, including S ones. Side and bottom 3D views displayed in Fig. 2D–I show how cell nuclei of MCF7 and MDA-MB-231 were found while crossing the mesh and inside the cages (see also 3D cuts in Figure S6). It is worth mentioning that some of these cells were observed while climbing the cages, although with no occurrence of the piling-up process observed for MCF10A (see orange arrows in Fig. 2).

### Invasion Assay Statistics

**Figure 3:**
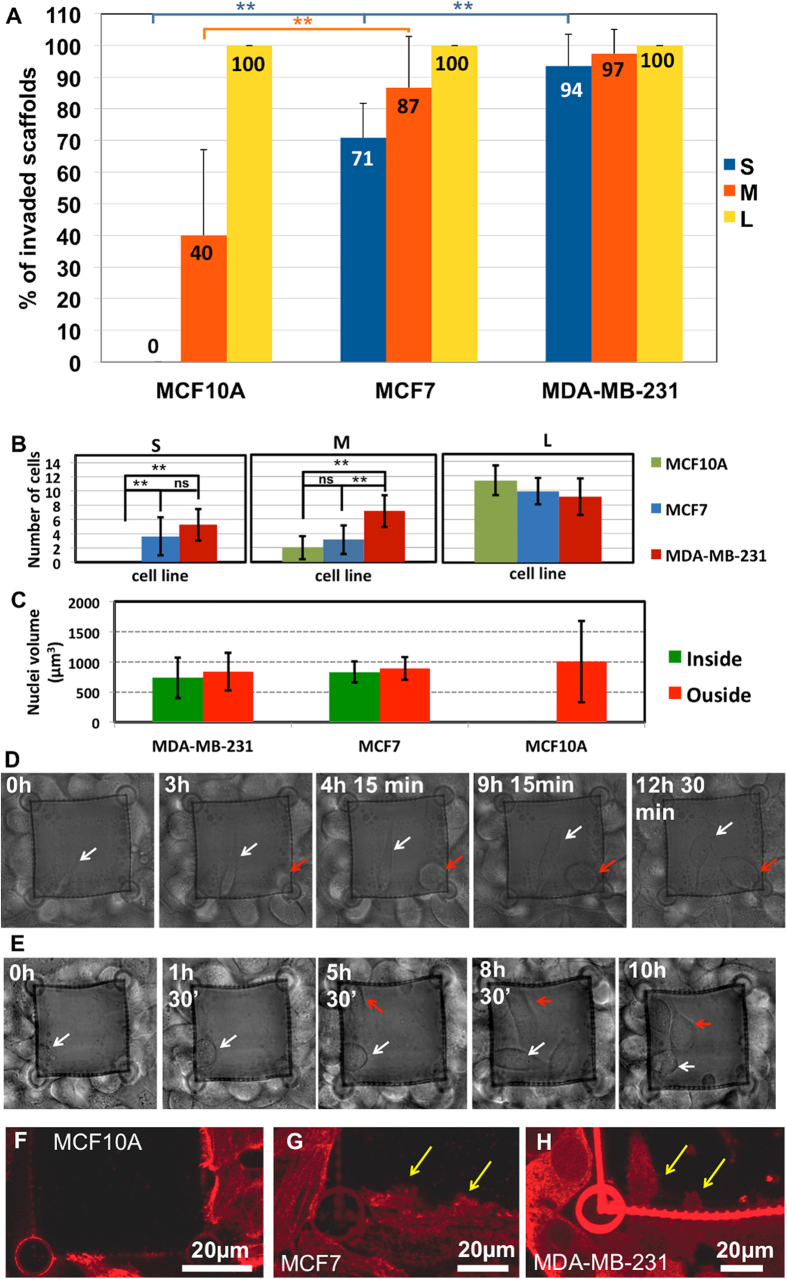
Invasiveness evaluation. ** Panel (A)** Histogram of the percentage of invaded scaffold for each pore area and cell line. The number of cages used to draw the histogram for S-size cage was n = 25, n = 38 and n = 28 for MCF10A, MCF7 and MDA-MB-231, respectively. In case of M-size cage, it was n = 16, n = 18 and n = 27 for MCF10A, MCF7 and MDA-MB-231, respectively. For L-size cage, it was n = 32, n = 23, and n = 25 for MCF10A, MCF7 and MDA-MB-231, respectively. * p < 0.05, ** p < 0.025 and ns p > 0.05, error bars indicate standard deviation. **Panel (B)** Histogram of the average number of cells inside invaded scaffolds. n = 8 for each histogram line. * p < 0.05, ** p < 0.025 and ns p > 0.05, error bars are standard deviations. **Panel (C)** Histogram of the average nuclear volume per cell line evaluated on confocal z-stack images of S-sized pore cages. Volumes were evaluated on n = 10 cells inside the cages and n = 10 cells outside the cages for MCF7 and MDA-MB-231 and n = 20 for MCF10A. Error bars indicate standard deviation. **Panels (D–E)** Representative frames of the time-lapse experiments with MDA-MB-231 and S-size cages (the corresponding movies are reported as Supplementary Movie 1 and 2, respectively). Red arrows indicate cells entering into the cage by exploiting one of the pillars, while white arrows indicate cells passing through the single pore without interacting with any of the pillars. **Panels (F–H)** Confocal microscope images of the three cell lines interacting with S-sized cages for MCF10A, MCF7 and MDA-MB-231, respectively. Fluorescence is obtained through immunostaining of myosin IIA as detailed in experimental section.

A statistical analysis was carried out to evaluate the percentage of invaded cages per cell line and per pore size (statistics on more than 230 cages, see experimental section for details). A cage was considered invaded when at least one stained nucleus was found within it. As detailed in the histogram in Fig. 3A, all investigated cell lines can always enter into L-sized structures. Tumor cells can easily pass through M pores (~87% for MCF7 and ~97% for MDA-MB-231), while MCF10A have some limitations in traversing them (only 40% of invaded M-size scaffolds). Statistical analysis reveals how the use of M pores (18 μm^2^ area) discriminates between the non tumoral cell line and the two tumorigenic cell lines, allowing for a first separation based on their aptitude to invade cage-like structures (p-value p = 0.0142 between MCF10A and MCF7 and p = 0.0009 between MCF10A and MDA-MB-231, t-test).

This MCF10A constraint in traversing the mesh monolayers was significantly enhanced for S size cages: we never found MCF10A into any of them. Remarkably, MCF7 and MDA-MB-231 were instead able to invade them (~71% and ~94%, respectively). This latter difference has been found to be statistically significant (p = 0.011, t-test on 5 independent experiments), letting us suggest that ~3 μm^2^ can be considered a suitable pore area to discriminate between each of the investigated cell lines. As shown in Fig. 3B, this is not possible by evaluating the average number of cells found into the invaded cages at the end of each experiment. To exclude any possibility that the S-size cages were only selecting for smaller cells, we have evaluated the average nuclear volume of cells inside and outside the structures and, as displayed in Fig. 3C, no significant differences were observed.

### Time lapse analysis

Since fluorescence microscopy gives a static picture of the experiment, in order to exclude any possible plastic deformation of the structure that would potentially promote cage invasion, we carried out time-lapse imaging on MDA-MB-231 (Supplementary Videos 1 and 2), the cell line having the highest aptitude to invade S-size cages. Despite the absence of any chemoattractant or surface treatment, typical 2D migration mechanisms on glass coverslip were observed, as suggested by the formation of many polarized protrusions (Figure S7) and by the migration speed of ~0.3 μm/min, in agreement with values already found for mesenchymal migration behavior^2^. Once the cells get in contact with the polymeric structure, they start to extend invadopodia into the pores. Cells can pass through the pores by interacting with the mesh and creating adhesion points to apply force, or by taking advantage of the large contact surface offered by the cylindrical structure of the pillars (white and red arrows, respectively, in Fig. 3D,E, see also Supplementary Videos 1 and 2). Importantly, we observed that in both circumstances the cage invasion process requires the cell to undergo strong deformation and pass through a single pore, as for the cells indicated by white arrows in Fig. 3D and red arrows in Fig. 3E (see also Figure S8 for a confocal image of a fixed sample).

All three cell lines show similar behavior during the early phases of interaction with the cages, in fact in Figure S5 it is possible to observe how MCF10A, MCF7 and MDA-MB-231 form long protrusions during the cage approaching phase and they also seem to exploit the same mechanisms of nuclear deformation (and translocation) into M and L cages. On the contrary MCF10A, MCF7 and MDA-MB-231 show different behaviors while interacting with S-sized cages. As displayed in Fig. 3F–H, long invadopodia-like protrusions trying to invade the cage (panel H) are observed in the case of MDA-MB-231 the cage, while for MCF7 several small invadopodia are formed at the cell-cage interface (panel G). This is not the case for MCF10A contacting S-sized cages, since in this case there are no detectable protrusions crossing the mesh and cell bodies simply lean against the cage (panel F).

### MCF10A and MDA-MB-231 Co-cultures

**Figure 4:**
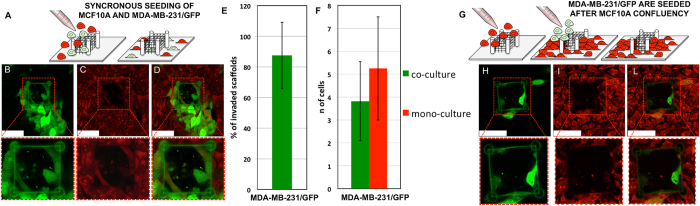
Co-culture experiments. ** Panel (A)** Schematic representation of the adopted synchronous seeding procedure, in which MCF10A and MDA-MB-231/GFP were seeded at the same time. **Panels (B, C)** Representative fluorescence confocal images of the S-size cages after the procedure schematized in panel (**A**) and after sample fixation and cell staining with Propidium Iodide. Panel B was acquired at green wavelengths, thus detecting signal only from MDA-MB-231/GFP, while panel C was acquired at red wavelengths, thus detecting signal from both cell lines. **Panel (D)** is the overlap between panels C and D. **Panel (E)** Percentage of invaded cages after 96 h of co-seeded MCF10A and MDA-MB-231/GFP at 10:1 concentration with the protocol displayed in panel A. Data are evaluated on three independent experiments and on a total of n = 15 S-cages. Error bar represents standard deviation. **Panel (F)** Average Number of MDA-MB-231/GFP inside S-sized cages after 96 h in co- and mono-cultures. Histogram shows the average number of cells invading a single cage in mono-culture (n = 10, red) and co-culture (n = 8, green) experiments. Error bars are standard deviation. **Panel (G)** Schematic representation of the adopted asynchronous seeding procedure, in which MDA-MB-231/GFP were seeded after 6 days of MCF10A culture, once confluency was obtained. **Panels (H, I)** Representative fluorescence confocal images of the S-size cages after the procedure schematized in panel G and after sample fixation and staining with Propidium. Panel H was acquired at green wavelengths, thus detecting signal only from MDA-MB-231/GFP, while panel G was acquired at red wavelengths, thus detecting signal from both cell lines. **Panel (L)** is the overlap between panels H and I. Scalebars in panels B-D, H-L represent 50 μm.

To evaluate the possibility of using this assay to detect the presence of MDA-MB-231 metastatic cells among a MCF10A population, two types of co-culture experiments were performed. In the first one, schematized in Fig. 4A, MDA-MB-231 stably expressing GFP and MCF10A were seeded at the same time, with MDA-MB-231/GFP being 1:10 of MCF10A (see experimental section for further details). After reaching the confluence point, the co-culture was fixed and stained with propidium iodide, thus obtaining red emitting MCF10A (co-localization of propidium was therefore observed for the GFP-expressing cells). As shown by the representative spectrally resolved confocal microscopy images reported in Fig. 4B–D for S-sized structures, only MDA-MB-231/GFP were found into the cages, while red emitting MCF10A cells create a thick epithelial-like layer all around the cages without traversing them. In three independent experiments we have found that on a total of 15 S-sized structures, 12 were invaded by MDA-MB-231, resulting in an invasion rate of ~0.88 (std ~0.22), as displayed in Fig. 4E. Statistical analysis on the number of invasion events (Fig. 4F) performed on three independent experiments reveal that the average number of MDA-MB-231/GFP found into S-sized cages is ~3.8 (std ~1.7, n = 11) in co-culture experiments, with comparison to an average of ~5.0 (std ~2.0, n = 8) MDA-MB-231 found into S-sized cages in mono-cultures after a fixed time (96 h). Since the t-test is not significant (p = 0.13), we can deduce that the presence of MCF10A does not affect the MDA-MB-231 invasion rate for S-sized cages.

As previously discussed, MCF10A mono-cultures result in the formation of 3D cell aggregates all-around the cages with S-sized pores. We carried out a proof-of-concept experiment in order to assess if the 3D cage-like structures can be reached and invaded by metastatic cells also when they are surrounded by a thick layer of non-invasive cells,. The experimental design is schematized in Fig. 4G: MDA-MB-231/GFP cells were seeded after 6 days with respect to MCF10A seeding, once cell aggregation was stably obtained (Fig. 2C). MDA-MB-231/GFP cells were 1:15 of estimated final MCF10A cells (see experimental procedures for further details). Also in this case, we found metastatic cells entering the S-sized cages (see Fig. 4H–L for confocal images and Figure S9 for a 3D reconstruction), even if MCF10A layer likely prevent a direct contact between MDA-MB-231 and the scaffolds. We assign this effect to the fact that MDA-MB-231 cells recognize and seem to be attracted by the 3D environment created by MCF10A cells aggregated against the cage. This is in agreement with a recent report by Ivers *et al.*^33^, in which MDA-MB-231 cells were shown to be attracted by spheroids of non-tumorigenic epithelial cells *in vitro*.

## Discussion

Here we propose cage-like microscaffolds, realized through two-photon lithography, which can be used as an invasion assay to discriminate between tumorigenic and non-tumorigenic human breast epithelial cell lines. The percentage of invaded S, M and L scaffolds (Fig. 3A) considerably increases as a function of the pore area for both MCF7 and MCF10A, with the MCF10A statistics being strongly dependent on pore size. This was not the case for MDA-MB-231, which invaded more than 90% of realized scaffolds regardless of the pore size. According to these results, under the conditions of our assay, the discrimination between non-tumorigenic and tumorigenic cells based on their invasive potential can be achieved with a 16 times smaller pore area than the one commonly used with a significantly higher degree of specificity^21^,^34^,^35^. Indeed, commercially available kits are usually based on a standard pore size of ~50 μm^2^ 21,36. As reported by Pellegrino *et al.*^21^, this type of assay does not discriminate between MCF7 and the MCF10A, resulting in a lack of selectivity. We demonstrate that M-sized cages allow a first discrimination between healthy MCF10A and the two tumorigenic cell lines, while a more accurate discrimination between MCF10A, MCF7 and metastatic MDA-MB-231 is possible with ~3 μm^2^ cage-like structures (with a zero invasion events found for MCF10A).

Both confocal microscopy analysis and time-lapse recording showed that cells converge from every direction toward the microcages, and that the entering process involves a pronounced deformation of the nucleus, depending on the cell type and the pore size. The here-observed trend could be related to cell deformability properties in terms of cell stiffness. Indeed, in many type of cancers, atomic force microscopy studies have highlighted that the Young’s Modulus of metastatic cells is remarkably lower than non-invasive ones^35^,^37^, including those used in our study^38^,^39^. This suggests that the nanomechanical properties of the cells contribute in determining their ability to traverse the mesh monolayers composing our cage-like structures. Moreover, the results on co-culture experiments suggest that invasive properties, in terms of aptitude in passing through 3 μm^2^ pores, are likely not affected by the presence of MCF10A, and that it is possible to detect an amount of metastatic cells within a population of non-invasive ones for starting concentration ratio as small as 1:10.

**Figure 5:**
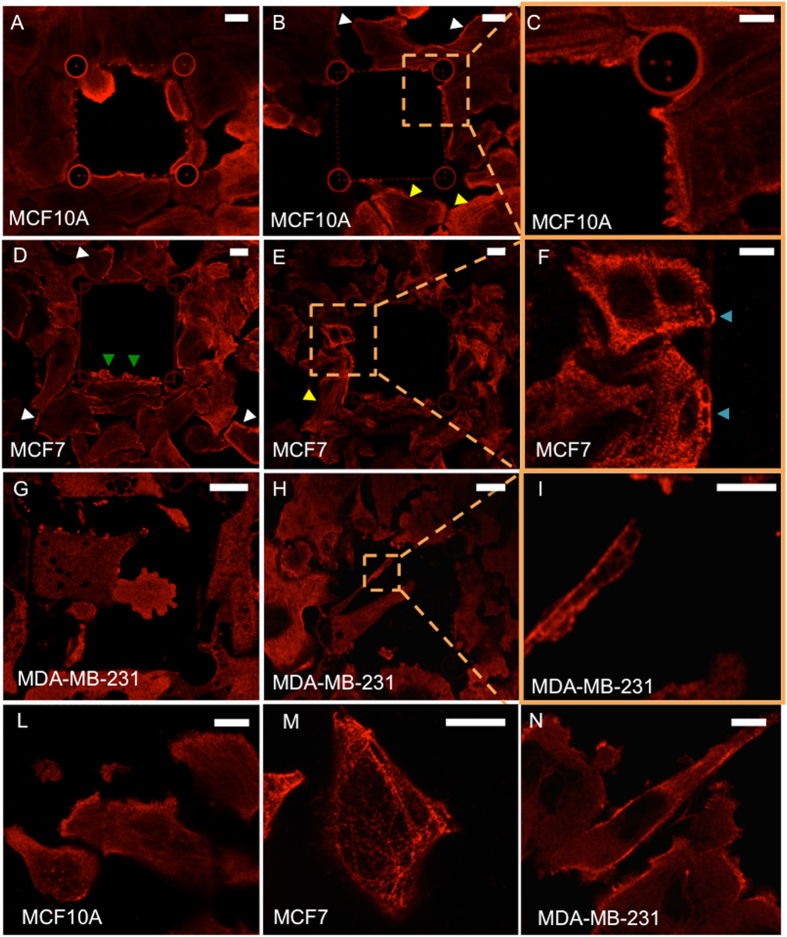
Confocal images of MCF10A, MCF7 and MDA-MB-231 cell lines stained for non-muscle myosin IIA light chain detection. ** Panel (A):** myosin localization in MCF10A cells entering in a M-sized cage-like structure. **Panels (B), (C):** myosin aggregates at the cell-cage interface for S pores. **Panel (D):** cortical myosin distribution of myosin IIA and small multiple myosin-rich invadopodia in MCF7 cells. **Panels (E), (F):** elongated myosin filaments and aggregates in MCF7 interacting with S pores. **Panels (G–I):** myosin distribution in MDA-MB-231/GFP cells interacting with a S-sized cage. In panel G a diffused myosin immunostaining is predominant. Panels H and I show small myosin aggregates in correspondence to invadopodia focal points. **Panels (L–N):** myosin IIA distribution in non-confluent samples. Scale bars represent 10 μm in panels A, B, D, E. G, H, L, M, and N, and 5 μm in panels C, F, I.

Since several literature reports have highlighted that migrating and invading cells undergo important cytoskeletal rearrangement, we evaluated myosin distribution in the three cell lines during interaction with the polymeric structures^28^,^29^,^30^,^31^. Immunofluorescence staining was performed as detailed in Methods section using anti non-muscle myosin IIA primary antibody. Typical fluorescence images for MCF10A, MCF7 and MDA-MB231 are reported in Fig. 5. In non-tumorigenic MCF10A cells it is possible to observe how non-muscle myosin is organized in long cables in the ventral area of the cell (yellow arrow heads in Fig. 5B), while, dorsally, a cortical distribution is predominant (white arrow heads in Fig. 5B). Even if MCF10A are not able to cross S-sized pores, in Fig. 5B and C it is possible to observe how myosin accumulates at the points where the cells contact the cage and apply force. In panel A myosin IIA aggregates are formed at the lamellipodium of an MCF10A invading an M-sized cage. MCF7 show similar myosin distribution if compared to MCF10A: ventrally located long fibers (Fig. 5E, yellow arrow heads), and cortical arrangements at dorsal areas (Fig. 5D, white arrow heads). Figure 5E and F also show myosin aggregates localized at the cell-cage interface (blue arrow heads in panel F) and myosin-rich small protrusions formed by the cells while attempting to invade the cage (Fig. 5D, green arrow heads). As regards MDA-MB-231, myosin filaments are poorly detectable both at the dorsal and ventral areas of the cells. In fact, especially in invading cells, myosin IIA seems to have a very disperse distribution (Fig. 5G) even if aggregates are found where invadopodia form focal adhesion points (Fig. 5 G–I). For reference, panels L-N of Fig. 5 show the myosin typical distribution of single mode migrating cells, with MCF10A and MCF7 showing a fibrillar distribution of myosin IIA and cortical distribution in MDA-MB-231 with poorly detectable myosin filaments.

The possibility to assess the myosin distribution on our assay, let us suggest that our polymeric structures are compatible with immunostaining protocols, widely adopted for analyzing most of cytoskeletal, nuclear and membrane markers. However, despite the high selectivity and versatility of our assay, one major limitation is the high fluorescence of the polymer, which can affect the analysis of fluorophores signals overlapping with the IP-L emission spectrum, mainly at blue and green wavelengths, as displayed in the sample emission spectra reporterd in Fig. S10. In contrast with standard transwell assay techniques, our system does not allow for estimating the percentage of cells succeeding in entering the cages. This is, however, intrinsically due to the fabrication and experimental design, since cells adhering far from the area in which cages are realized have a lower probability to interact with the scaffolds with respect to those close to them. On the other hand, this test is conceptually different from other approaches used to evaluate tumorigenesis, mainly based on cells proliferation, since it is based on the aptitude of cells to deform their body and traverse very small pores along the three axes, strongly related to cells mechanical properties.

In summary, this work reports the fabrication of new and fully biocompatible polymeric 3D structures and their use as invasion assay to study cell aptitude to pass through mesh monolayers having very small pore size compared to cell nuclei. The statistical analysis shows how it is possible to use sufficiently small structures (~3 μm^2^ pore size in this case) to discriminate between tumorigenic and non-tumorigenic human breast cells, suggesting the specificity of this *in vitro* assay for identifying MCF7 and MDA-MB-231 with respect to MCF10A. This allows defining a threshold pore area for mesh monolayers below which non-tumorigenic cells are not allowed to enter into the cage. Time-lapse analysis and confocal microscopy results also suggest that all investigated cell lines can reorganize their cytoskeleton and deform their nucleus to pass through a single pore, with MDA-MB-231 showing the peculiar mechanical characteristics of traversing 3 μm^2^ pores in more than 90% of the realized cages. Since MCF10A cells were never found into S-sized cages, this approach results in an innovative, powerful assay to directly discriminate between non-tumorigenic and tumorigenic human breast cancer cell lines with high degree of specificity.

## Methods

### 3D structures fabrication and characterization

Micrometric structures were fabricated by a commercially available two photon DLW system (Photonic Professional, Nanoscribe GmbH) equipped with a femtosecond-pulsed laser at 780 nm focused into a droplet of IP-L photoresist (Nanoscribe GmbH) by means of a high-numerical aperture oil-immersion objective (N.A. = 1.4). Photoresist polymerization properties were investigated at laser powers ranging from 4 mW to 16 mW. Optimized cage-like structures consisting of four cylindrical pillars and aligned ridges were thus polymerized on glass using a laser power of 13 mW for pillars and 11 mW for ridges at a scan speed of 30 μm/sec, resulting in in-plane thickness of ~0.5 μm and out-of-plane thickness ~1 μm . Mesh-grids were written at 10 μm, 5 μm or 2.5 μm periodicity. The sample was then immersed for 1h at room temperature (RT) in SU-8 Developer (MicroChem) and preserved in isopropyl alcohol until use. Isopropyl alcohol was substitute by phosphate saline buffer (PBS) and PBS was substitute by cell growth medium. Realized samples were dried only occasionally to perform SEM inspections.

### Cell cultures

Three different human epithelial cell lines were investigated. (i) MCF10A cells (non-tumorigenic epithelial cell line derived from human mammary gland ATCC CRL®-10317™) were cultured in 1:1 DMEM:F12 medium (Gibco) supplemented with 5% horse serum (Invitrogen), 2 mM L-Glutamine (Sigma Aldrich), 20 ng/ml EGF (Sigma Aldrich), 0.5 mg/ml hydrocortisone (Sigma Aldrich), 100 ng/ml cholera toxin (Sigma Aldrich), 10 μg/ml insulin (Sigma Aldrich), 1% PenStrep (100 U/ml of penicillin, 100 μg/ml streptomycin, Gibco). (ii) MCF7 cells (derived from primary tumor, human invasive breast ductal carcinoma ATCC® HTB-22™) were grown in DMEM medium (Gibco) supplemented 10% FBS (Gibco) + 2mM L-Glutamine (Sigma Aldrich), 1% PenStrep. (iii) MDA-MB-231 (human breast adenocarcinoma cell line derived from a metastatic site ATCC® HTB-26™) were cultured in RPMI medium supplemented 10% FBS (Gibco) + 2mM L-Glutamine (Sigma Aldrich), 1% PenStrep. MDA-MB-231/GFP Cell line (CELL BIOLABS, INC.) were grown in DMEM (high glucose), 10% FBS, (Sigma Aldrich), 2 mM L-glutamine, 1% Non Essential Amino Acids, 1% PenStrep. All cell lines were cultured at 37 °C and 5% CO_2_ and split every three days 1:5 by using trypsin/EDTA (Sigma Aldrich).

### Migration/invasion experiments

Depending on the cell type, confluence point was reached after 48 hours or 96 hours, starting from a concentration of 10^5^ cells/ml. Cells were fixed in 4% paraformaldehyde for 20 min at RT and then washed with PBS. A solution of Triton X-100 0.5% was added for 5 min at RT and multiple PBS washings were done. Nuclei were then stained with 4’,6-diamidino-2-phenylindole (DAPI) 1 ug/mL for 10 min. For the statistical analysis the total amount of analyzed structures for S-size cages was: n = 25, n = 38 and n = 28 for MCF10A, MCF7 and MDA-MB-231, respectively. In case of M-size cages it was n = 16, n = 18 and n = 27 for MCF10A, MCF7 and MDA-MB-231, respectively. For L-size cages it was n = 32, n = 23, and n = 25 for MCF10A, MCF7 and MDA-MB-231, respectively.

For the co-culture experiments MDA-MB-231 stably expressing green fluorescent protein (GFP) cell line was used (MDA-MB-231/GFP). In the first experiment (Fig. 4A–F) they were co-seeded with MCF10A at a concentration of 1.4*10^5^ cells/ml and 1.4*10^4^ cells/ml, respectively, and stained after 5 days. In the second experiment (Fig. 4G–L) MCF10A were cultured to reach confluency. After 6 days cells formed acini-like structures over the cages and MDA-MB-231/GFP were added at a concentration of 0.5*10^5^ cells/ml. Co-cultures were grown in MDA-MB-231/GFP and MCF10A culture media 1:1. Cell staining was performed after 6 days of co-culture (4% paraformaldehyde for 20 min at RT, Triton X-100 0.5%5 min at RT, propidium iodide 4 μg/ml for 5 min at RT). To perform anti non-muscle myosin immunostaining cells were fixed with parafolmaldehyde for 15 min at RT and permeabilized with Triton X-100 0.5% in PBS for 1 min. Samples were treated for 1 h at RT with BSA 2% in PBS and incubated for 1 h at RT with anti non-muscle myosin IIA primary antibody from ABCAM (1:100). The secondary antibody was AlexaFluor 568 from Life Technologies (1:150). After each steps samples were washed three times for 5 min.

### Confocal Microscope Analysis and Time Lapse

To visualize DAPI stained nuclei, confocal laser scanning microscopy (CLSM) using a Leica TCS SP8 confocal microscope was performed. Images were acquired with the LasAF Software using a 63X oil-immersion objective (HC PL APO CS2 63X/1.40 OIL, Leica Germany). The pinhole was set at 1 Airy unit (95 μm). A 405 nm continuous diode laser was used for sample excitation with a power of 2.5 mW. Fluorescent emission was detected in the spectral window between 420 nm and 500 nm by a GaAsP hybrid photodetector (Leica HyD™). 3D reconstructions were made in order to enlighten the spatial distribution of cells within and around structures.

To perform time-lapse analysis, the Leica TCS SP8 confocal microscope was equipped with an incubator to preserve cell growth conditions. Bright-field images were acquired with the transmission photomultiplier tube (PMT). 180 μm × 180 μm wide images were acquired at 1024 × 1024 pixels with a pixel size of 176 nm × 176 nm, scan speed of 200 Hz. Z-stacks were acquired with a z-step size of 1 μm for a total z-volume of 60 μm to cover the entire thickness of the cage. 3D reconstruction was performed with the 3D View module of the LasAF Software^40^,^41^,^42^.

The co-culture images were obtained by detecting two different spectral ranges simultaneously. The GFP-labelled cells were observed by a hybrid photodetector detecting photons between 500 and 550 nm. The propidium-stained cells were detected in the wavelength range 580–710 nm. In this case, the cells were excited by a femtosecond pulsed white light laser (SuperK™ from NKT Photonics), centered at 488 nm (repetition rate 80 MHz).

Cell migration speed has been calculated by analyzing the cell body displacement in time-lapse videos (540s between frames, image 180 × 180 microns).

## Additional Information

**How to cite this article**: Spagnolo, B. *et al.* Three-dimensional cage-like microscaffolds for cell invasion studies. *Sci. Rep.* **5**, 10531; doi: 10.1038/srep10531 (2015).

## Supplementary Material

Supplementary Video 1

Supplementary Video 2

Supplementary Information
