# Supplementary material for: Three-dimensional cage-like microscaffolds for cell invasion studies
Source: Sci Rep. 2015 May 27;5:10531. doi: 10.1038/srep10531 (PMC4650598; doi:10.1038/srep10531)
Supplement: Supplementary Information [file srep10531-s3.pdf]

Three-dimensional cage-like microscaffolds for cell invasion studies

Barbara Spagnolo, Virgilio Brunetti, Godefroy Leménager, Elisa De Luca, Leonardo Sileo, Teresa Pellegrino, Pier Paolo Pompa, Massimo De Vittorio and Ferruccio Pisanello

SUPPLEMENTARY MATERIAL

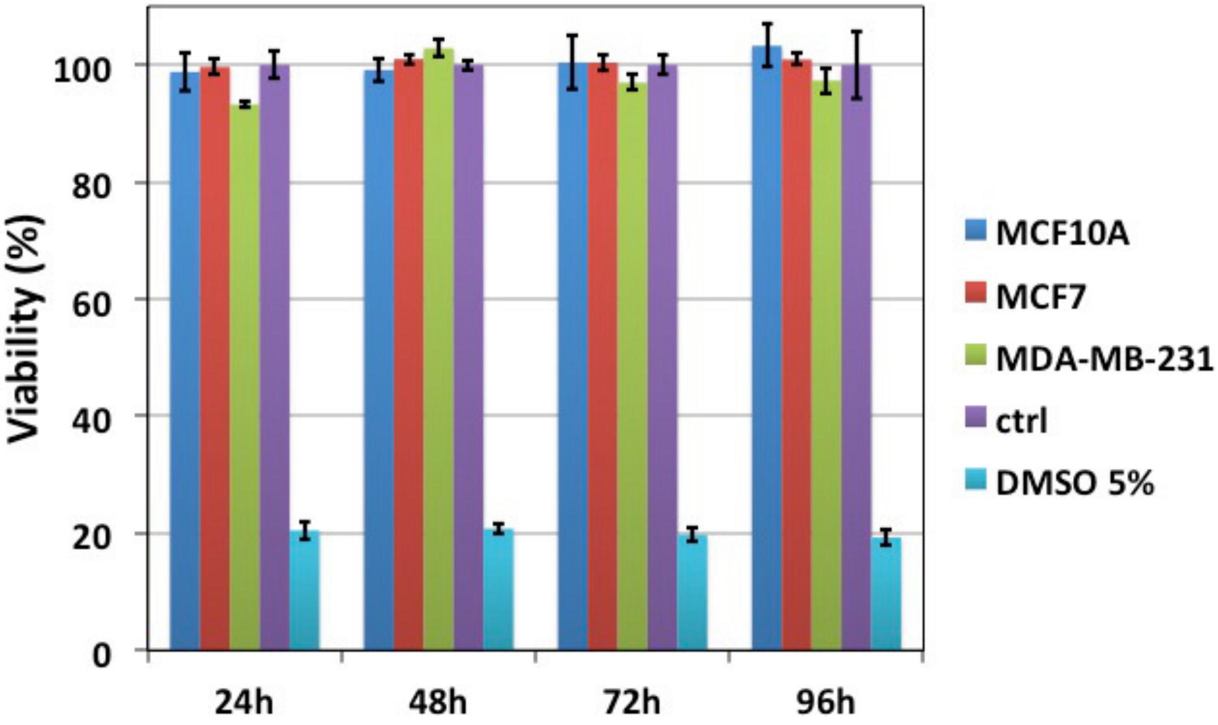

Figure S1. Cells Viability on IP-L photoresist for all the investigated cell lines. Values are mean ± SD. ctrl represents the positive controls; MCF10A cell line was chosen as representative positive control; similar values were obtained also for MCF7 and MDA-MB-231 (data not shown). Negative controls (DMSO 5%) were treated with 5% DMSO displaying a strong viability decrease (c.a 80%) with respects to the positive controls; MCF7 cell line was chosen as representative negative control; similar values were obtained also for MCF10A and MDA-MB-231 (data not shown). Results were analyzed by Two-way ANOVA and values compared to the control by the Bonferroni post-hoc test. Differences between treated samples and controls ($n \geq 4$) were considered statistically significant for $*P < 0.05$, and non-significant for $P > 0.05$. Errorbars represent standard deviations.

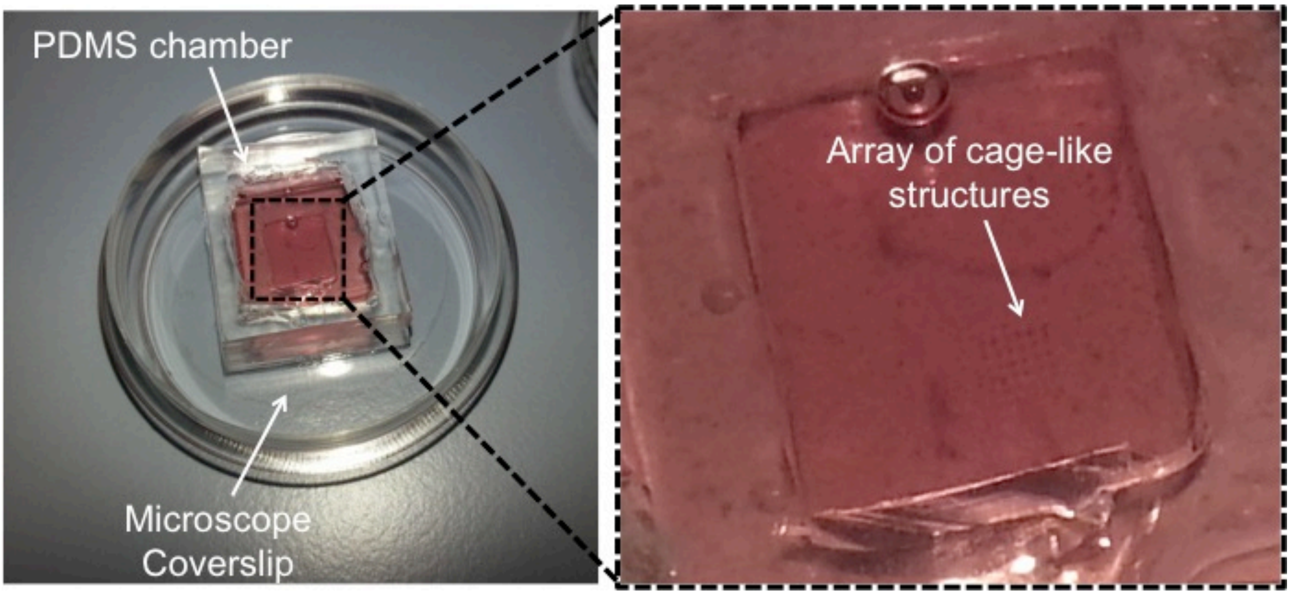

Figure S2. Picture of the specimen just after cell seeding. In the zoom on the right panel it is possible to distinguish the array of cage-like structures.

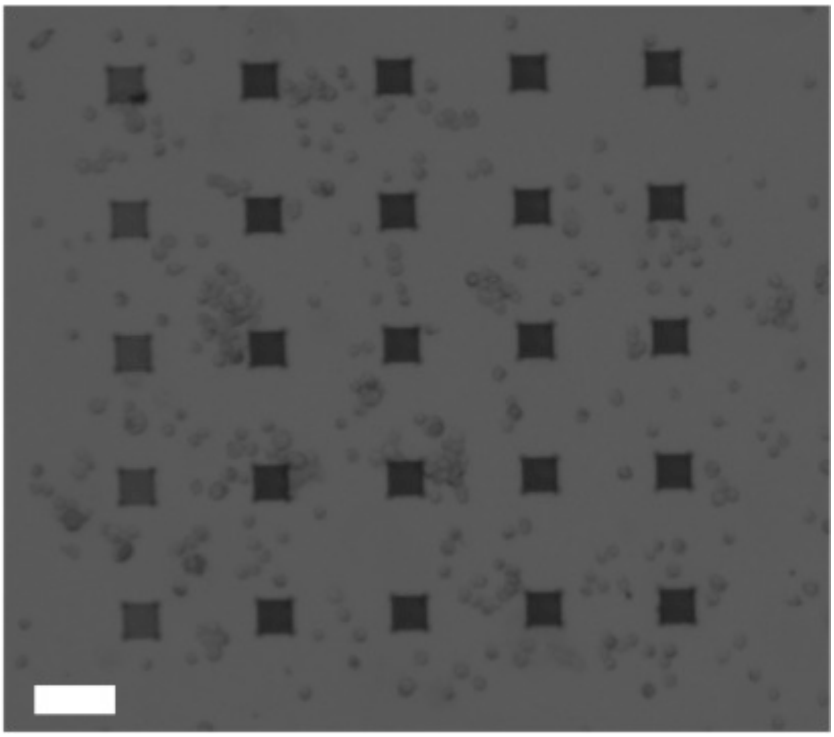

Figure S3. Bright field microscope image of an array of cage-like structures just after MDA-MB-231cell seeding. Scale bar represents 100µm.

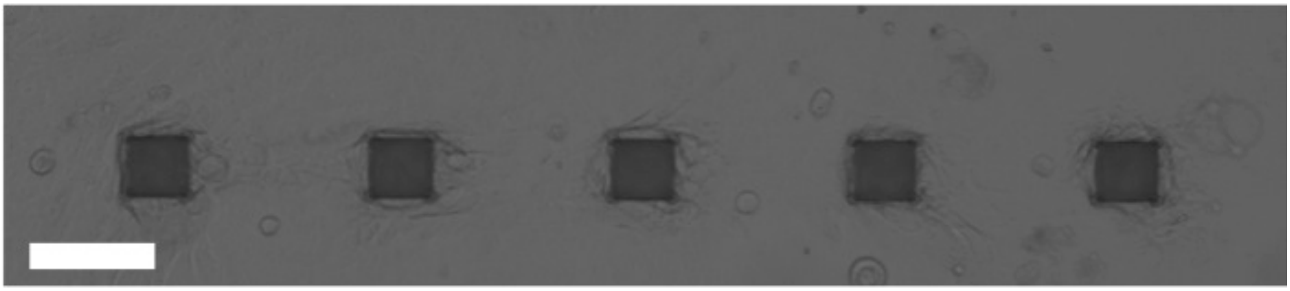

Figure S4. Bright field microscope image of 5 cage-like structures when confluency of MCF10A was reached. Scale bar represents 100µm.

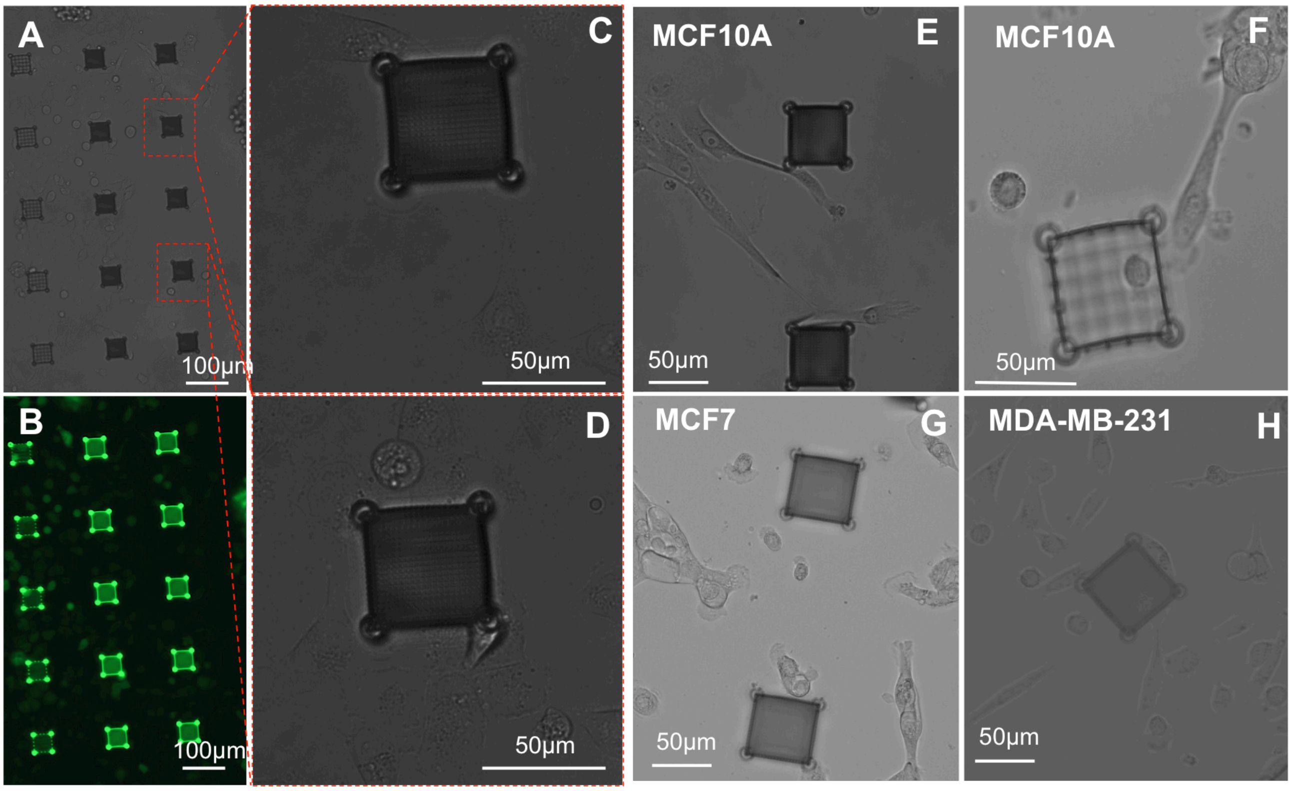

Figure S5. Cells seeded at low concentration approaching the structures 24h after seeding. (A-D) MDA-MB-231/GFP, (E,F) MCF10A, (G) MCF7, (H) MDA-MB-231.

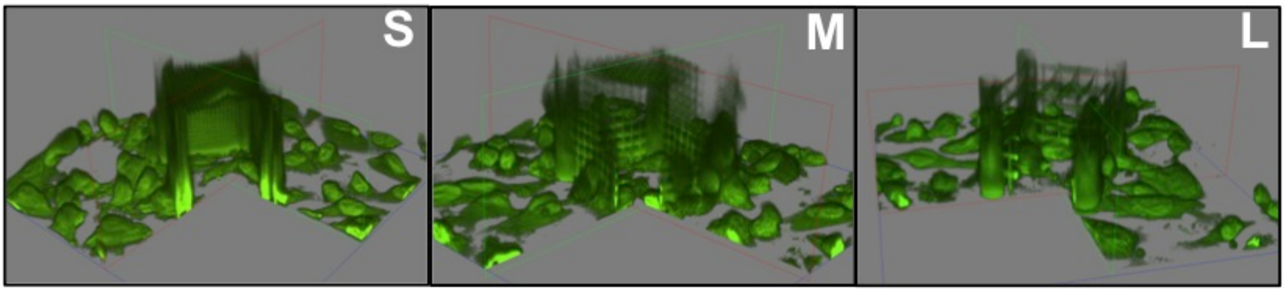

Figure S6. Section cuts of invaded S, M and L cage-like structures. Internal views show the spatial disposition of the DAPI-stained MDA-MB-231 and their adhesion to the substrate.

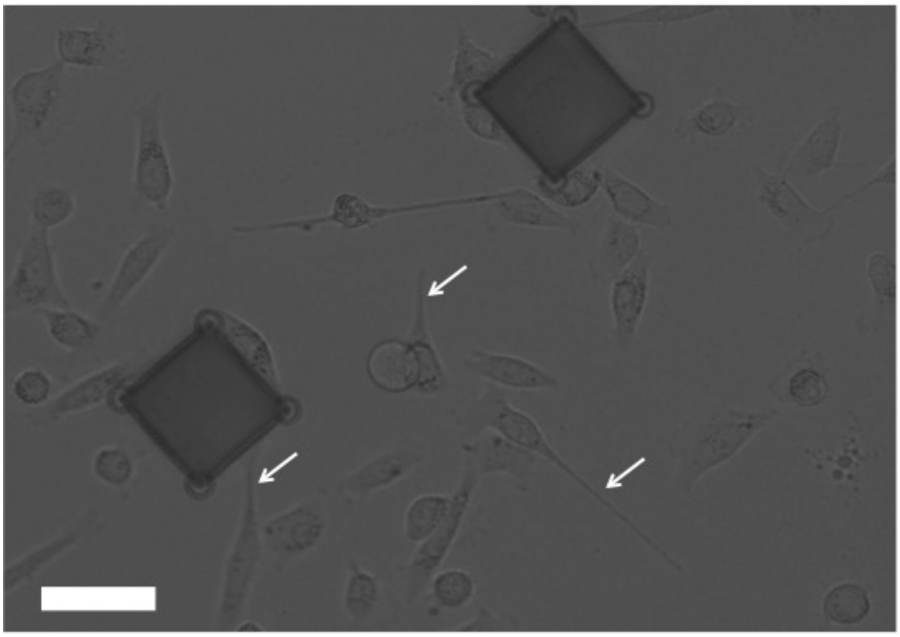

Figure S7. MDA-MB-231 cells approaching cage-like structures. White arrows indicate cells emitting long protrusions. Scale bar represents 50μm.

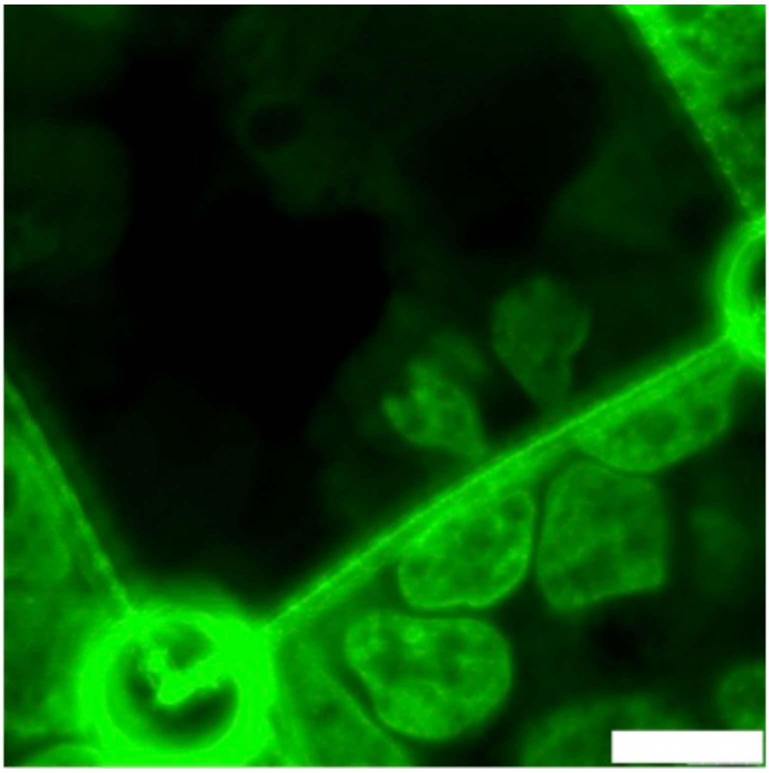

Figure S8. MDA-MB-231 nuclei caught while passing through a single pore in S cage-like structure. The image is acquired in false color and collected after sample fixation and DAPI staining. Scale Bar represents 10μm.

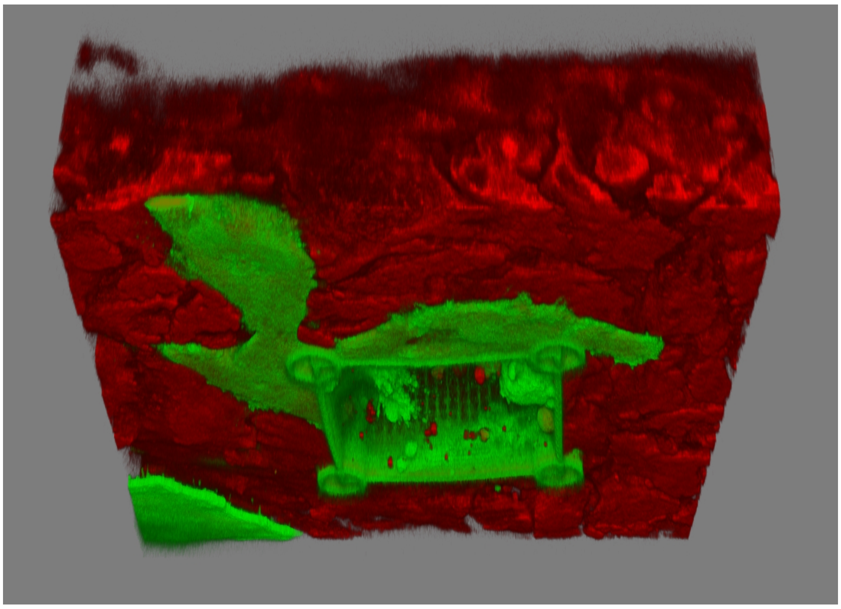

Figure S9. 3D reconstruction of a MDA-MB-231/GFP and MCF10A coculture. MDA-MB-231/GFP were seeded after MCF10A reached confluency following the method schematized in Figure 4B. The image shows how metastatic cells can crawl towards (and invade) the cage by displacing the thick MCF10A layer .

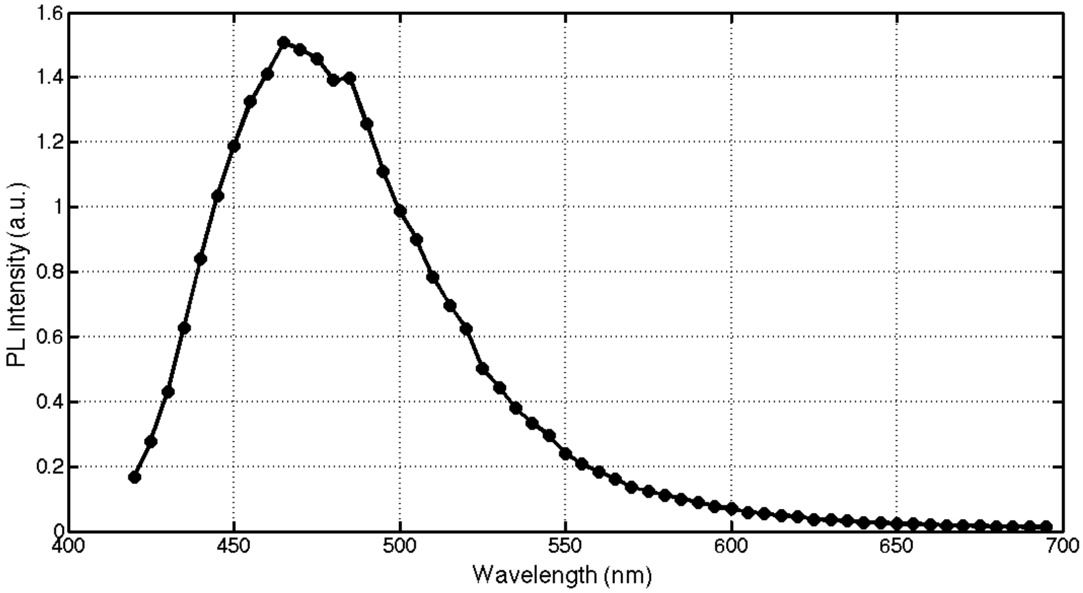

Figure S10. Emission spectra of cage-like a structure (excited at 405nm).
